# Midgut malrotation first presenting as acute bowel obstruction in adulthood: a case report and literature review

**DOI:** 10.1186/1749-7922-6-22

**Published:** 2011-07-29

**Authors:** Okiemute F Emanuwa, Abraham A Ayantunde, Tony W Davies

**Affiliations:** 1 Department of Surgery, Queen Mary's Hospital, Frognal Avenue, Sidcup, DA14 6LT, London, UK

**Keywords:** Midgut malrotation, acute abdomen, Ladd's bands, computed tomography scan, laparoscopy

## Abstract

Malrotation of the midgut is generally regarded as paediatric pathology with the majority of patients presenting in childhood. The diagnosis is rare in adults, which sometimes leads to delay in diagnosis and treatment. A high index of suspicion is therefore required when dealing with patients of any age group with abdominal symptoms. We present a case of a 55-year old man who presented with an acute abdomen with preoperative computed tomography scan and operative findings confirming midgut rotation. The duodenum, small bowel, caecum and appendix were abnormally located, with the presence of classical Ladd's bands. There was no evidence of intestinal volvulus. The patient underwent an emergency laparotomy with an uneventful postoperative recovery.

A review of the literature is presented to highlight the rarity of intestinal malrotation and the controversies surrounding its management in the adult population.

## Introduction

Midgut malrotation is a congenital anomaly in the embryological development of the foetal intestinal rotation. It has been estimated that it affects approximately 1 in 500 live births [1]. However, the true incidence is difficult to determine as a substantial number of cases will go undetected throughout life. The vast majority of the complications associated with midgut malrotation present in the first month of life and 60-85% of cases are diagnosed in this age group [1,2]. It is reported that more than 90% of patients will present by the time of their first birthday [3]. Adult midgut malrotation is very rare and its incidence has been reported to be between 0.0001% and 0.19% [3,4]. Most adult diagnoses of midgut malrotation are made in asymptomatic patients; either on imaging investigations for unrelated conditions or at operations for other pathology. This scenario of incidental diagnosis is becoming increasingly common, particularly with improvements, and increased use, of diagnostic imaging techniques in modern practice. However, there are a small proportion of affected adults who may present with acute or chronic symptoms of intestinal obstruction or intermittent and recurrent abdominal pain. The true diagnosis in this age group is fraught with immense difficulty, especially because the typical presentation is with non-specific symptoms and the fact that adult Surgeons usually have low index of suspicion and may not consider the diagnosis a possibility in the initial evaluation of adult patients with abdominal pain.

We report a case of an adult patient with an acute presentation of midgut malrotation which highlights the dilemmas of preoperative diagnosis, as supported by a review of the literature.

## Case report

A 55-year old gentleman was admitted to the Accident and Emergency department with a three day history of acute onset, cramp like abdominal pain. There was associated nausea but no vomiting. Bowels had been opened the day before admission but no flatus had been passed for 24 hours. There were no other associated red flag symptoms. The patient had never experienced similar symptoms and had no previous medical or surgical history.

On examination, the patient was afebrile and haemodynamically stable. The abdomen was moderately distended with significant tenderness in the central, epigastric and left hypochondrial regions. There was no evidence of peritonitis.

Routine admission blood tests including serum electrolytes, urea, amylase, lactate, liver function tests (LFTs), clotting profile, C-reactive protein (CRP) and an arterial blood gas (ABG) were normal. However, a full blood count (FBC) demonstrated a haemoglobin level of 14.2 g/dl with a slightly raised total white cell count (WCC) of 10.3 × 10^9 ^and a neutrophilia of 7.0 × 10^9^.

**Figure 1:**
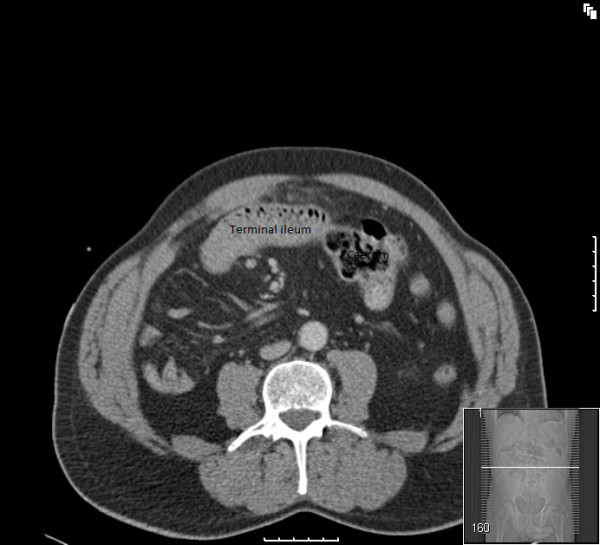
**CT scan showing caecum on the left side of the abdomen and terminal ileum entering the caecum from the right side**.

**Figure 2:**
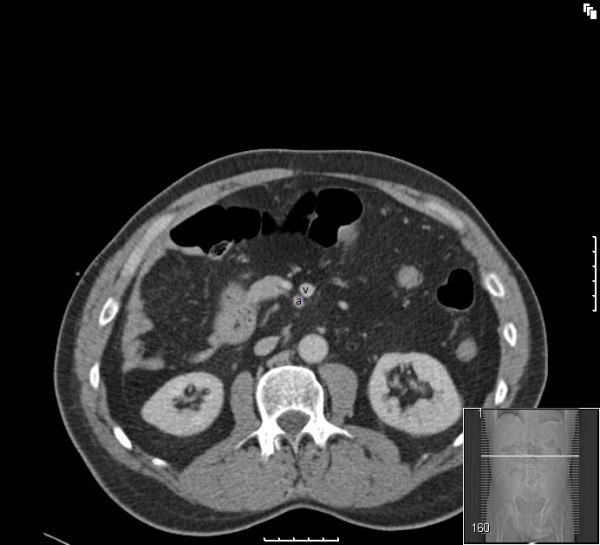
**CT scan showing inverse relationship of SMA to SMV (a-artery and v-vein)**.

**Figure 3:**
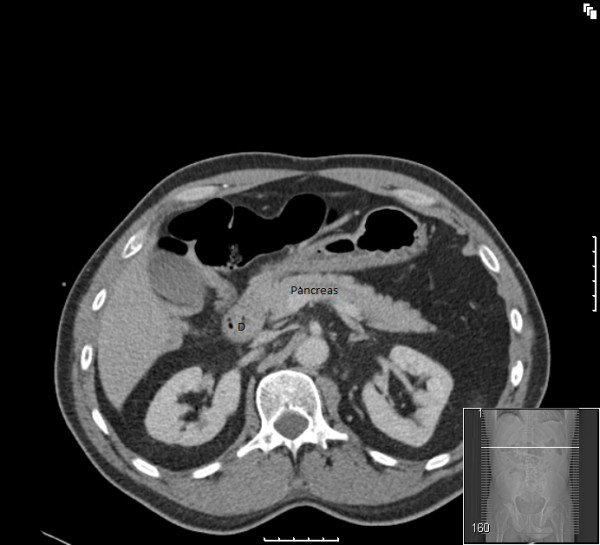
**CT scan showing lack of progression of the duodenum across the aorta and the spines (D-duodenum)**.

**Figure 4:**
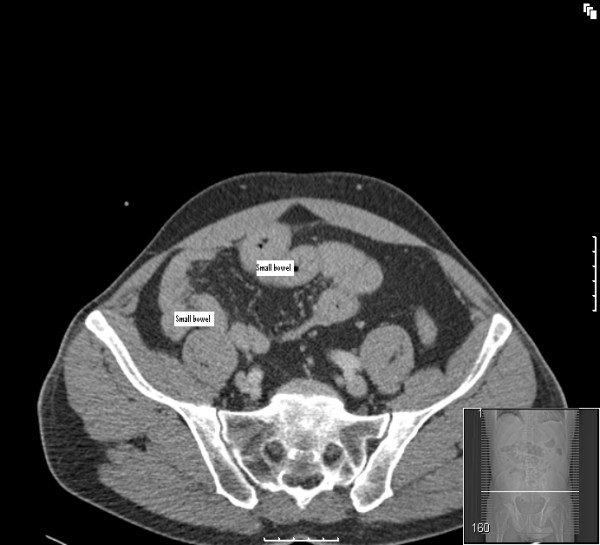
**CT scan showing most of the small bowel on the right side of the abdomen**.

A chest radiograph did not reveal air under the diaphragm. Abdominal radiograph showed non-dilated gas filled loops of bowel in the central and upper abdominal regions. The diagnosis remained elusive until an emergency computed tomography (CT) scan (Figures 1, 2, 3, 4) was obtained which demonstrated features of malrotation. The duodenum was malpositioned below and to the right of the ascending colon and hepatic flexure. The caecum was located in the left upper quadrant. There were also a few dilated loops of small bowel in the upper abdomen.

**Figure 5:**
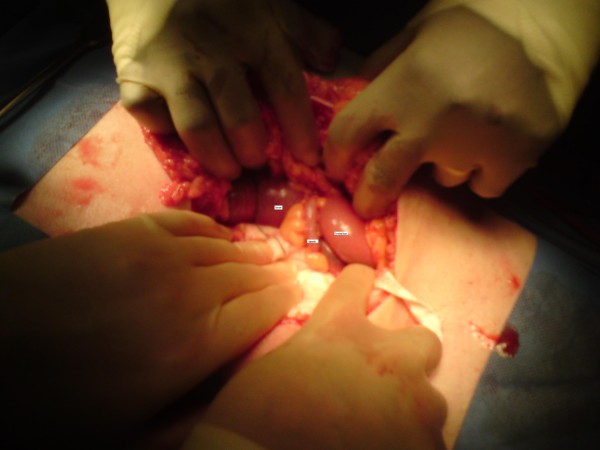
**Photograph showing high caecum and appendix located on the left side of the abdomen**.

**Figure 6:**
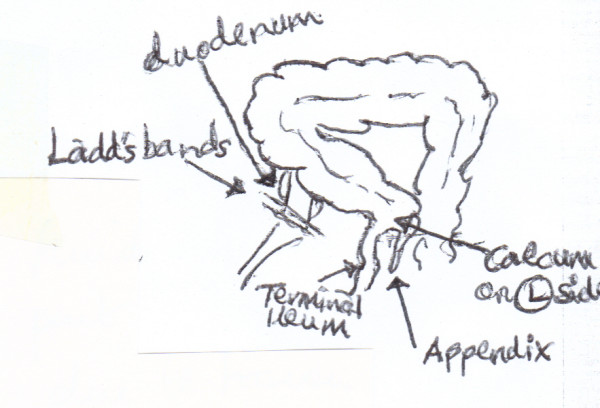
**Graphical representation of the intra-operative findings**.

The patient was resuscitated with intravenous fluids, analgesia and prepared for an emergency exploratory laparotomy. The findings at operation included dilated small bowel in the upper abdomen, partial torsion and necrosis of the greater omentum, the caecum was on the left side of the abdomen tethered by torted omentum, and loops of small bowel occupying the right paracolic gutter and the right iliac fossa. There were fibrous bands over the distal part of the duodenum, on the right side of the abdomen, confirming midgut malrotation (Figures 5 &6).

The twisted, necrotic omentum was excised, the congenital bands were divided and an appendicectomy was carried out. The anatomical malrotation was left uncorrected.

The patient had an uneventful postoperative recovery and was discharged home on the fifth day post- surgery. On follow up he was well and there had been no late complications. He had returned to his premorbid level of function and did not report any symptom recurrence.

## Discussion and review of the literature

Initial presentation of symptomatic midgut malrotation is rare in adults. However, a significant number of cases remain quiescent during childhood. Incidental diagnosis may then occur in adulthood; when imaging investigations are carried out for other symptoms or, during surgery for unrelated pathology. It has been reported that the incidence of malrotation in adults is approximately 0.2%. However, it is probable that this rate will rise with future developments in diagnostic imaging. It is difficult to ascertain the true incidence, but evidence from post mortem studies suggest that gut malrotation may affect up to 1 in 6000 [3,4].

Midgut malrotation is broadly considered a deviation from the normal 270 degree counterclockwise rotation of the gut during embryonic development. During week 4 of foetal development, the embryonic gut, consisting of a straight endodermal tube, develops vascular pedicles to be divided into the foregut, midgut and hindgut based on the anatomical blood supply. The midgut is supplied by the superior mesenteric artery (SMA) and by the fifth week of embryonic life, it begins a process of rapid elongation and outgrows the capacity of the abdominal cavity. This leads to a temporary physiological herniation into the umbilical cord at about the sixth week of life with return to the abdominal cavity about 4 to 6 weeks later. During this period, the midgut undergoes a 270 degree counterclockwise rotation around the SMA axis. This process leads to the formation of the duodenal C-loop, placing it behind the SMA in a retroperitoneal position and emerging at the ligament of Treitz. The progressive reduction of the physiological midgut herniation commences at about week 10 of embryonic development. The duodeno-jejunal flexure (DJF) and jejunum to reduce first and lie to the left. The distal small bowel then follows and lies progressively to the right of the abdominal cavity. The descent of the caecum from its higher position in the right upper quadrant forms the latter part of this complex rotational development; it becomes positioned in the right lower abdomen. The ascending colon then assumes a retroperitoneal position, also on the right side. The base of the small bowel mesentery subsequently fuses with the posterior peritoneum in a diagonal fashion, from the ligament of Treitz at the DJF to the caecum, completing the whole process at about the eleventh week of foetal development [1,4-6].

The failure of the normal physiological rotation of the midgut leads to various degrees of anomaly including the entire small bowel remaining on the right side of the abdomen, the caecum, appendix and colon on the left and an absent ligament of Treitz. In addition, the small bowel mesentery may develop a narrow vertical attachment and the peritoneal fibrous bands fixing the duodenum and caecum to the abdominal wall may persist. These congenital bands extend from the right lateral abdominal wall, across the duodenum and attach to the undescended caecum and are known as Ladd's bands [2,4,6,7]. Ladd's bands compress the duodenum and can potentially cause duodenal obstruction. The malrotation of the gut and abnormal location of the caecum produces a narrow superior mesenteric vascular pedicle, as opposed to the normally broadbased small bowel mesentery. This narrow SMA takeoff and lack of posterior peritoneal fusion predispose to subsequent midgut volvulus and obstruction with potential vascular catastrophe [7,8].

Midgut malrotation in adults presents in numerous ways and the symptoms are non-specific. The clinical diagnosis in adolescents and adults is difficult because it is rarely considered on clinical grounds. Many patients remain asymptomatic and the diagnosis is discovered incidentally during investigations or laparotomy for other unrelated problems in adult life. Wang and Welch [4] showed that 24 of 50 patients were clinically asymptomatic in their case series of adolescents and adults with malrotation. Adults with a rotational abnormality of the gut usually present differently to paediatric patients. Two distinct patterns of adult presentations have been reported in the literature: acute and chronic [5,7,9]. Chronic presentation is more common in adults. This is characterised by intermittent crampy abdominal pain, bloating, nausea and vomiting over several months or years. The symptoms may be highly nonspecific. However, the range of clinical presentations, underlines the need for a high index of suspicion of midgut malrotation, when investigating the cause of intermittent and varying abdominal symptomatology in a healthy young adult [5,7]. Dietz et al [5] studied a series of 10 adults with bowel obstruction caused by intestinal malrotation. They reported that 5 adults presented with chronic features and that the duration of symptoms extended to 30 years. Fu et al [7] reported that 6 of 12 patients in their series presented with chronic intermittent abdominal symptoms. Diagnostic delays are common in this group of patients because of the nonspecific nature of the presentations. The pathophysiology of these chronic symptoms may relate to the compression effect of Ladd's bands running from the caecum and ascending colon to the right abdominal wall [5,10].

The other group of symptomatic adults typically present with symptoms of acute bowel obstruction and these patients may or may not report a previous history of abdominal symptoms, as with our patient. These patients may on occasion, have symptoms and signs of an impending abdominal catastrophe. Moldrem et al [9] reported that 48.5% of their thirty-three patients presented with an acute abdomen. Acute presentation may be due to volvulus of the midgut or ileocaecum, reported as the most common cause of bowel obstruction in adults with gut malrotation. Other causes of acute presentation may be related to internal herniation caused by Ladd's bands. There is also a subgroup of acutely presenting adult patients with malrotation. They are identified when affected by other common abdominal diseases. Their unusual intestinal anatomy results in atypical signs and symptoms. These patients may present with localised peritonitis in the right upper quadrant or on the left side of the abdomen if their appendix becomes inflamed. The atypical presentations may lead to confusion, as one common abdominal pathology may mimics another, leading to incorrect diagnosis of conditions such as acute appendicitis, cholecystitis, pancreatitis, perforated peptic ulcer disease and left colonic diverticulitis. Several authors have reported observing atypical presentations of this nature before discovering gut malrotation with abnormal location of the caecum and appendix at surgery [5,7].

We can expect an increase in the incidental diagnosis of gut malrotation with increasing and widespread use of radiological investigations. Diagnostic features of midgut malrotation can be identified using plain abdominal radiograph, ultrasound scan (USS), computed tomography (CT) scan, magnetic resonance imaging (MRI) scan and mesenteric arteriography [9,11]. Conventional plain radiography is neither sensitive nor specific in the diagnosis of gut malrotation although right-sided jejunal markings and the absence of a stool-filled colon in the right lower quadrant may be suggestive, leading to further investigation. Abdominal colour Doppler USS may reveal malposition of the SMA, raising the suspicion of gut malrotation with or without the abnormal location of the hollow viscus [9,11,12]. Characteristic USS findings of midgut volvulus were first described by Pacros et al and include duodenal dilatation with distal tapering and fixed midline bowel and mesentery twisted around the SMA axis. These features classically present as the 'whirlpool' sign [13]. The reported gold standard for diagnosis of gut malrotation is an upper gastrointestinal (UGI) contrast study, particularly in the paediatric age group [5,11,12]. This will generally show the duodenum and duodenojejunal flexure located to the right of the spine. The use of a contrast enema in conjunction with the UGI study has also been advocated as it can be used to demonstrate an abnormally located ileocaecum and right colon. However, contrast study findings may be nonspecific and a normal study does not exclude the possibility of gut malrotation [5,7,10,11].

CT scan with or without UGI contrast study is increasingly used preferentially as it is now considered the investigation of choice; providing diagnostic accuracy of 80% [5,9,11]. CT and MRI scans may show the SMV to be in an anomalous position; posterior and to the left of the SMA. In addition, they may show the abnormal anatomical arrangements of the midgut with the duodenum not crossing the spine. Deviation from the normal positional relationship of SMV and SMA was originally described by Nichols and Li [14] as a useful indicator of the diagnosis of midgut malrotation. However, abnormal orientation of the SMA-SMV relationship is not entirely diagnostic of malrotation; it can also be seen in some patients without the pathology and a proportion of patients with malrotation may have a normal SMA-SMV relationship [11]. Patients with gut malrotation will often have an underdeveloped or absent uncinate process of the pancreas. This is possibly due to the failure of the SMA to migrate to the left of the SMV [9,11]. The CT appearance of midgut volvulus is diagnostic of malrotation. The shortened mesentery allows the small bowel and mesentery to twist and wrap around the narrowed SMA pedicle to create a distinctive 'whirlpool' appearance on CT scan. This pattern was first described by Fisher in a patient with midgut volvulus [15]. It can be detected with both abdominal USS and CT scan. CT scan findings of gut malrotation and small bowel obstruction without volvulus, may show internal herniation secondary to Ladd's bands.

Mesenteric angiography was previously used but is now rarely indicated in the evaluation of malrotation. It has the capacity to demonstrate the abnormal relationship between, and detect the patency of, the mesenteric vasculature. Angiography was used to demonstrate the characteristic corkscrew appearance of a whirling SMA and its branches; the 'barber pole sign' as well as extensive collaterals caused by proximal SMA occlusion [16]. However, its role has been superseded by the CT scan which has the overall advantage of not only detecting the abnormal location of the midgut but also the reversed mesenteric anatomical relationship as well as any other intra-abdominal anomalies associated with malrotation.

Symptomatic midgut malrotation undoubtedly requires surgical intervention although the management of asymptomatic patients is more controversial. Choi et al [17] reviewed 177 patients over a 35-year period. They found that asymptomatic patients had a low risk of intestinal volvulus and therefore advised that routine investigations, screening and elective surgery were not necessary with close follow-up. However, it is increasingly argued that all suitable patients with intestinal malrotation should undergo surgical correction regardless of age as it is impossible to predict which patients will develop catastrophic complications [8]. Several small case series have recommended that elective Ladd's procedure should be performed in all patients with intestinal malrotation. The authors of the studies that include cases of life threatening small bowel ischaemia argue this point particularly strongly [3,5,7,9]. Of course, the operative policy should be based on the presentation and suspected diagnosis; the potential risks of the procedure need to be weighed against the benefits.

The surgical management of intestinal malrotation was first described by William Ladd in 1936 [6] and this remains the mainstay of treatment. The classical Ladd's Procedure consists of 4 parts: division of Ladd's bands overlying the duodenum; widening of the narrowed root of the small bowel mesentery by mobilising the duodenum and division of the adhesions around the SMA to prevent further volvulus; counterclockwise detorsioning of the midgut volvulus if present and appendicectomy to prevent future diagnostic dilemma of an abnormally located appendix [6]. The original Ladd's procedure was described for the paediatric population group and the full components of this procedure may not be offered in the adult group [4-6,9]. Most authors are of the opinion that Ladd's procedure is an adequate treatment for intestinal malrotation. Fu et al [7] reported a complete resolution of symptoms in 9 and near complete resolution in 2 of 11 patients. Dietz et al [5] also reported a complete symptomatic resolution in 8 of their 10 patients treated surgically. Variations of the technique used to manage intestinal malrotation have been introduced to prevent recurrent volvulus. These include re-establishment of the normal gut anatomy by duodenopexy, caecopexy and suture fixation of the ascending colon to the right abdominal wall, in the retroperitoneal position [4,5,18]. We offered a modified procedure to our patient by performing a division of Ladd's bands and an appendicectomy. There was no volvulus and we did not feel that the duodenum needed to be mobilised and straightened in this case. Our patient has been completely symptom free during 12 months of follow up.

There are recent reports of the use of the laparoscopic approach in the surgical treatment of intestinal malrotation. The technique appears to be safe and effective when performed by experienced laparoscopic surgeons, especially in the absence of volvulus [2,7,8,18,19]. Laparoscopic Ladd's procedure in paediatric groups is increasingly reported in the literature. It is becoming more accepted as an initial approach to surgical correction of intestinal malrotation, resulting in shorter hospital stays. There are few reports of this approach in adults. The laparoscopic approach can be technically challenging and conversion to open procedure is common [2,7,8,19]. A few published works have indicated that the laparoscopic approach can be successful in patients with malrotation and midgut volvulus [8,19]. A retrospective analysis of both open and laparoscopic Ladd's procedures by Stanfill et al performed at the Children's Hospital of Illinois, USA noted that short-term results were superior with the laparoscopic approach and can be achieved without any increase in the duration of the operation [20].

## Conclusions

Intestinal malrotation is a rare condition but is considered an important cause of bowel obstruction in adults. The diagnosis of malrotation after childhood is difficult and usually not readily considered as the cause of intra-abdominal symptoms. The presentation is usually nonspecific and this often leads to diagnostic and treatment delay with possible bowel ischaemia and necrosis. Evidence of which portends a poor prognosis and death. Therefore, a high index of suspicion needs to be maintained and prompt surgical intervention must be considered in order to prevent an abdominal catastrophe and fatality. There are no reliable means of identifying which group of patients with intestinal malrotation will develop subsequent complications. In the light of this, many authors are now advocating early surgical intervention in the form of a standard and modified Ladd's procedure. There is evidence in the literature that the use of Ladd's procedure or ordinary division of Ladd's bands and adhesiolysis relieves symptoms and in fact, prevents recurrence in the majority of patients.

## Patient's consent

Written informed consent was obtained from the patient for publication of this case report and accompanying images. A copy of the written consent is available for review by the Editor-in-Chief of this journal.

## Abbreviations

ABGs: Arterial blood gases; CRP: C-reactive protein; CT: Computed tomography; DJF: Duodenal jejunal flexure; FBC: Full blood count; LFTs: Liver function tests; MRI: Magnetic resonance imaging; SMA: Superior mesenteric artery; USS:Ultrasound scan; WCC: White cell count.

## Competing interests

The authors declare that they have no competing interests.

## Authors' contributions

OFE was involved in postoperative care, conceived the write up, performed the literature search and manuscript preparation.

AAA performed the operation with TWD, involved in the preoperative and postoperative care, conceived the write up, performed the literature search and manuscript preparation.

TWD performed the operation with AAA, involved in the preoperative and postoperative care, conceived the write up, performed the literature search and manuscript preparation.

All authors read and approved the manuscript for submission.
